# Berbamine promotes macrophage autophagy to clear *Mycobacterium tuberculosis* by regulating the ROS/Ca^2+^ axis

**DOI:** 10.1128/mbio.00272-23

**Published:** 2023-06-29

**Authors:** Su Zhang, Xuefeng Zhou, Min Ou, Xiangdong Fu, Qiao Lin, Xiaoyu Tao, Zhaoqin Wang, Aimei Liu, Guobao Li, Yuzhong Xu, Guoliang Zhang

**Affiliations:** 1 National Clinical Research Center for Infectious Diseases, Guangdong Provincial Clinical Research Center for Tuberculosis, Shenzhen Third People’s Hospital, Southern University of Science and Technology, Shenzhen, China; 2 Guangzhou Medical University, Guangzhou, China; 3 Department of Clinical Laboratory, The Baoan People’s Hospital of Shenzhen, The Second Affiliated Hospital of Shenzhen University, Shenzhen, China; 4 Department of Tuberculosis, Guangxi Chest Hospital, Liuzhou, China; University of Calgary, Calgary, Canada

**Keywords:** *Mycobacterium tuberculosis*, berbamine, autophagy, ROS, calcium

## Abstract

Drug-resistant tuberculosis (TB) poses a major threat to global TB control; consequently, there is an urgent need to develop novel anti-TB drugs or strategies. Host-directed therapy (HDT) is emerging as an effective treatment strategy, especially for drug-resistant TB. This study evaluated the effects of berbamine (BBM), a bisbenzylisoquinoline alkaloid, on mycobacterial growth in macrophages. BBM inhibited intracellular *Mycobacterium tuberculosis* (Mtb) growth by promoting autophagy and silencing ATG5, partially abolishing the inhibitory effect. In addition, BBM increased intracellular reactive oxygen species (ROS), while the antioxidant *N*-acetyl-L-cysteine (NAC) abolished BBM-induced autophagy and the ability to inhibit Mtb survival. Furthermore, the increased intracellular Ca^2+^ concentration induced by BBM was regulated by ROS, and BAPTA-AM, an intracellular Ca^2+^-chelating agent, could block ROS-mediated autophagy and Mtb clearance. Finally, BBM could inhibit the survival of drug-resistant Mtb. Collectively, these findings provide evidence that BBM, a Food and Drug Administration (FDA)–approved drug, could effectively clear drug-sensitive and -resistant Mtb through regulating ROS/Ca^2+^ axis-mediated autophagy and has potential as an HDT candidate for TB therapy.

**IMPORTANCE:**

It is urgent to develop novel treatment strategies against drug-resistant TB, and HDT provides a promising approach to fight drug-resistant TB by repurposing old drugs. Our studies demonstrate, for the first time, that BBM, an FDA-approved drug, not only potently inhibits intracellular drug-sensitive Mtb growth but also restricts drug-resistant Mtb by promoting macrophage autophagy. Mechanistically, BBM activates macrophage autophagy by regulating the ROS/Ca^2+^ axis. In conclusion, BBM could be considered as an HDT candidate and may contribute to improving the outcomes or shortening the treatment course of drug-resistant TB.

## INTRODUCTION

Tuberculosis (TB), an infectious disease caused by *Mycobacterium tuberculosis* (Mtb) infection, is a leading cause of death worldwide. Globally in 2020, there were an estimated 1.3 million deaths among human immunodeficiency virus (HIV)-negative people and an additional 214,000 deaths among HIV-positive people, which was an increase compared with the previous year (1). Moreover, the presence of drug-resistant TB, including multiple drug-resistant TB (MDR-TB) and extensively drug-resistant TB (XDR-TB), means there is a need for more effective drugs and novel treatments. For this purpose, host-directed therapy (HDT), which modulates host immune responses to improve pathogen eradication, has emerged as a promising area of research to fight TB (2).

Berbamine (BBM), a bisbenzylisoquinoline alkaloid, is a natural, potent, pharmacologically active biomolecule isolated from the shrub *Berberis amurensis*. BBM modulates different cell-signaling pathways (JAK/STAT, CAMKII/c-Myc) in various cancers (3), is effective as an anti-diabetic supplement (4), and, more recently, was reported to inhibit the entry of SARS-CoV-2 (severe acute respiratory syndrome coronavirus 2) virus, the causative agent of COVID-19, into host cells (5). However, there are few studies on how BBM regulates mycobacteria.

Our study demonstrates, for the first time, that BBM potently inhibits intracellular mycobacterial growth and that this effect is mediated by BBM enhancing autophagy in infected macrophages. Further research showed that cytoplasmic reactive oxygen species (cROS) and mitochondrial ROS (mROS) production were upregulated by BBM, which ultimately enhanced autophagy in infected macrophages. Moreover, BBM treatment increased cytoplasmic Ca^2+^ and mitochondrial Ca^2+^ levels, and ROS could regulate this ability. In addition, BBM could also inhibit drug-resistant Mtb. These findings suggest that BBM modulates macrophage functions to restrict intracellular mycobacterial growth. BBM may be a potential HDT drug candidate for TB treatment.

## MATERIALS AND METHODS

### Cell culture

Human monocytic cell line THP-1 was purchased from the National Collection of Authenticated Cell Cultures, China, and maintained in RPMI 1640 medium (Gibco, 11875093) supplemented with 10% fetal bovine serum (Gibco, 10091148), 0.05 mM 2-mercaptoethanol (Sigma, M3148), 1 mM sodium pyruvate (Gibco, 11360-070), and 1% penicillin–streptomycin (Gibco, 15140122). Cells were cultured in a humidified incubator at 37°C and 5% CO_2_. In infection experiments, no antibiotic was used.

### Bacterial culture

Drug-resistant TB were collected from patients who were diagnosed by minimum inhibitory concentration (MIC) assay in the clinical laboratory. The INH-resistant TB had mutations in nucleotides 944 and 1388 of *katG* gene (KatG S315T, R463L). The RFP-resistant TB had a mutation in nucleotide 1592 of *rpoB* gene (RpoB S531L). H37Rv-green fluorescent protein (H37Rv-GFP) was constructed in our laboratory. All of the drug-resistant TB strains, H37Rv-GFP and standard strains H37Rv, were cultured in Middlebrook 7H9 broth (BD Biosciences, 271310) supplemented with 10% Oleic Acid-Dextrose-Catalase (OADC) (BD Biosciences, 212240), 0.5% glycerol, and 0.05% Tween 80 at 37°C for 1–2 weeks to achieve mid-logarithmic phase (optical density at 600 nm [OD_600_] ≈ 0.8). Cultures were harvested, resuspended in phosphate-buffered saline (PBS) with 0.05% Tween 20, 25% glycerol, and stored at −80°C. One vial of the stock was thawed to calculate CFU per milliliter. On the day of infection, mycobacteria were thawed, washed, and sonicated before use.

### Preparation of BMDMs

BMDMs were prepared from femurs and tibiae of wild-type C57BL/6J mice. Cleaned bones were cut and flushed to collect bone marrow with a 1-mL syringe, and the collected bone marrow suspension was lysed with ACK lysing buffer (Gibco, A1049201) to remove red cells and plated in RPMI 1640 supplemented with 25 ng/mL macrophage colony stimulating factor (M-CSF) (Peprotech, 315-02-100). After 5 days of culture, the cells were detached from the dishes with Trypsin-EDTA (Gibco, 25200072), resuspended in fresh RPMI 1640, and plated in appropriate plates.

### Drug administration

The chemicals BBM (MedChemExpress, HY-N0714), BAPTA-AM (MedChemExpress, HY-100545), and rifampicin (MedChemExpress, HY-B0272) were dissolved in dimethyl sulfoxide (DMSO), while 3-MA (MedChemExpress, HY-19312), NAC (Selleck, S1623), CQ (Cell Signaling, 14774s), and Isoniazid (MedChemExpress, HY-B0329) were dissolved in double-distilled water. All solutions were aliquoted and stored at −20°C or −80°C.

### Cell viability assay

THP-1 cells were seeded at 5 × 10^4^ cells/well in a 96-well plate in RPMI 1640 medium and differentiated using phorbol 12-myristate 13-acetate (PMA; Sigma, P8139) at 100 ng/mL for 24 hours, followed by 24-hour rest in media without PMA. Different concentrations of drugs were added and cultured for an additional 48 hours, and CCK-8 reagent (Vazyme, A311-02) was added into the well (10 µL/well) and incubated at 37°C for 2 hours to measure cell viability. The absorbance was detected at 450 nm with a Varioskan LUX Multimode Microplate Reader (Thermo Fisher, Varioskan LUX Multimode Microplate Reader). The cell viability rate (%) of three independent experiments was calculated as follows:


$$Cell\;viability\;rate\;\left(\%\right)=\frac{OD\;of\;treated\;cells-OD\;of\;background}{OD\;of\;control\;cells-OD\;of\;background}\;\times \;100\%$$

CC_50_ values were calculated using a four-parameter logistic curve (GraphPad Prism 7.0).

### Mtb infection and enumeration of CFUs

THP-1 cells were seeded at 2 × 10^5^ cells/well in a 24-well plate and pretreated with relative drugs for 24 hours. The cells were infected with Mtb strains H37Rv at an MOI of 10 for 4 hours, then washed three times with prewarmed sterile PBS to remove extracellular bacteria, and cultured with RPMI 1640 medium at 37°C and 5% CO_2_. After 4 and 48 hours, cells were lysed with PBS containing 0.1% SDS, and the lysates were gradient diluted on Middlebrook 7H10 agar (BD Biosciences, 262710) supplemented with 10% OADC, 0.5% glycerol, and incubated vertically at 37°C for 2–3 weeks. Bacterial colonies were counted, and CFUs were estimated per dilution.

### H37Rv-GFP-infected cell analysis by flow cytometry

THP-1 cells were seeded at 2 × 10^5^ cells/well in a 24-well plate and pretreated with relative drugs for 24 hours. The cells were infected with H37Rv-GFP at an MOI of 10 for 4 hours. Then washing three times with prewarmed sterile PBS to remove extracellular bacteria and cultured with RPMI 1640 at 37°C and 5% CO_2_ for 48 hours. Cells were collected in fluorescence activated cell sorting (FACS) tubes, and the percentages of GFP-positive cells were measured by flow cytometry (BD Biosciences, Canton II) after 4-hour and 24-hour infection and analyzed by FlowJo X 10.0.7 according to the manufacturer’s protocol.

### Measurement of ROS

THP-1 cells were seeded at 2 × 10^5^ cells/well in a 24-well plate and pretreated with relative drugs for 24 hours. The cells were infected with H37Rv at an MOI of 10 for 4 hours. Extracellular bacteria were removed by washing three times with prewarmed sterile PBS and cultured with RPMI 1640 at 37°C and 5% CO_2_ for 24 hours. According to the manufacturer’s instructions, the generation of cROS was quantified using apocynin (Sigma, 309800). The generation of mROS was quantified using MitoSOX red mitochondrial superoxide indicator (Invitrogen, M36008). The cells were incubated with apocynin (10 µM) and MitoSOX (5 µM) for 15 minutes at 37°C in the dark. All fluorescence intensities of cells were measured by flow cytometry (BD Biosciences, Canton II) and analyzed by FlowJo X 10.0.7 according to the manufacturer’s protocol.

### Measurement of Ca^2+^ concentration

The THP-1 cells were seeded at 2 × 10^5^ cells/well in a 24-well plate and pretreated with relative drugs for 24 hours. The cells were infected with H37Rv at an MOI of 10 for 4 hours. Extracellular bacteria were removed by washing three times with prewarmed sterile PBS and cultured with RPMI 1640 at 37°C and 5% CO_2_ for 24 hours. The level of cytoplasmic Ca^2+^ (cyto-Ca^2+^) was measured by the fluorescent probe Fluo-4 AM (MedChemExpress, HY-101896), the level of mitochondrial Ca^2+^ (mito-Ca^2+^) was measured by the fluorescent dye Rhod-2 AM (MedChemExpress, HY-D0989), and the level of endoplasmic reticulum Ca^2+^ (ER-Ca^2+^) was measured by the fluorescent probe Mag-Fluo-4 AM (AAT Bioquest, 20401). The cells were incubated with Fluo-4 AM (5 µM), Rhod-2 AM (5 µM), and Mag-Fluo-4 AM (5 µM) for 15 minutes at 37°C in the dark. All fluorescence intensities of cells were measured by flow cytometry (BD Biosciences, Canton II) and analyzed by FlowJo X 10.0.7 according to the manufacturer’s protocol.

### LC3 puncta analysis

mRFP-GFP-LC3 (microtubule associated protein 1 light chain 3) reporter THP-1 macrophages (5 × 10^5^ cells/well) were differentiated using PMA in 2-cm coverglass bottom dishes and pretreated with relative drugs for 24 hours. The cells were infected with H37Rv (MOI of 10:1) for 4 hours, then washed three times in PBS, and kept cultured for 24 hours. Cells were fixed in 4% paraformaldehyde solution (Beyotime, P0099) for 15 minutes at room temperature and then stained with DAPI (Beyotime, C1002). Images were acquired with a Zeiss LSM700 confocal microscope and processed using ZEN software. About 15–20 infected cells were analyzed, and the level of autophagy was measured by enumerating the number of LC3 puncta per cell.

### RNA interference and transfection

THP-1 cells were transfected with autophagy related 5 (ATG5) small interfering RNA (siRNA) (RiboBio, stB0003079A) and negative control siRNA (RiboBio, siN0000001) using Lipofectamine RNAiMAX (ThermoFisher, 13778075) according to the manufacturer’s protocol. After 24 hours, cells were differentiated by PMA and seeded at 2 × 10^5^ cells/well in a 24-well plate.

### Western blot

Cells were harvested and lysed in RIPA lysis buffer (Beyotime, P0013B) for 5 minutes on ice. The protein concentration of the resultant lysates was measured with a bicinchoninic acid protein kit (Beyotime, P0010S). Equal amounts of protein from each sample were separated by SDS-PAGE and electron-blotted on PVDF membranes. The membrane was blocked with 5% skim milk powder solution in PBS with Tween 20 for 2 hours at room temperature and incubated with primary antibodies overnight at 4°C. The membranes were incubated with relevant secondary antibodies at room temperature for 1 hour and visualized using an ECL detection solution (Beyotime, P0018AS). The digital images of the protein bands were acquired using a ChemiDoc MP imaging system (Bio-Rad, ChemiDoc MP). The primary antibodies used in the present study were anti-LC3 (Sigma, L8918), anti-GAPDH (CST, 4970L), and anti-ATG5 (CST, 12,994T).

### Statistical analysis

All the presented data and results were confirmed in at least three independent experiments. The data were represented as the mean ± SD and analyzed using GraphPad Prism 7.0 software (GraphPad, San Diego, CA, USA). Statistical significance was analyzed by one-way analysis of variance (ANOVA) or two-way ANOVA. ∗*P* < 0.05; ∗∗*P* < 0.01; ∗∗∗*P* < 0.001; ∗∗∗∗*P* < 0.0001.

## RESULTS

### Berbamine suppresses intracellular mycobacterial replication in macrophages

**Fig 1:**
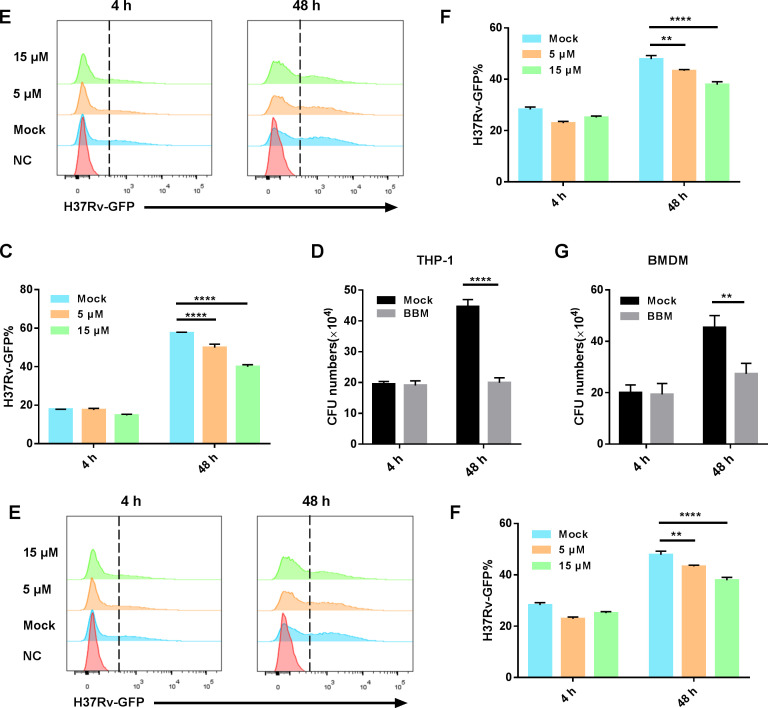
Berbamine suppressed intracellular mycobacterial growth in THP-1 cells and bone marrow–derived macrophages (BMDMs). Dose-dependent cytotoxicity and CC_50_ of berbamine in THP-1 cells (**A**). THP-1 cells were differentiated by PMA (100 ng/mL) for 24 hours, while BMDMs were prepared from mouse rear leg bone marrow. Cells were then pretreated with the doses of berbamine 24 hours before infection with H37Rv-GFP (MOI 10:1) and analyzed by flow cytometry at 4-hour and 48-hour post-infection. Representative flow cytometry images of H37Rv-GFP-positive cells were captured, and the percentage of cells positive for GFP was calculated using Flow Jo software in THP-1 cells (**B and C**) and BMDMs (**E and F**). CFU counts from THP-1 cells and BMDMs treated with or without berbamine (15 µM) after 4- and 48- hour infection with *M. tuberculosis* H37Rv (**D and G**). NC, negative control. Data represent means ± SDs for three independent experiments. Two-way analysis of variance (ANOVA) was performed in (C, D, F, and G). **P < 0.01; ***P < 0.001; ****P < 0.0001.

First, the cytotoxicity of BBM was verified by the CCK-8 reagent, and the CC_50_ was determined as 43.66 µM in Tohoku Hospital Pediatrics-1 (THP-1)-differentiated macrophages (Fig. 1A). Subsequently, the ability of BBM to suppress the intracellular growth of mycobacteria *in vitro* was tested. THP-1-differentiated macrophages were pretreated and maintained with DMSO, 5 and 15 µM BBM for 24 hours, then were infected with an H37Rv fluorescent reporter strain that expresses green fluorescent protein (GFP) (H37Rv-GFP, the multiplicity of infection [MOI] 10:1), and were analyzed by flow cytometry at 4 and 48 hours post-infection, respectively. BBM did not significantly impact the phagocytosis rate at 4 hours after infection, but 5 and 15 µM of BBM treatment for 48 hours after infection significantly reduced the survival of intracellular H37Rv-GFP (Fig. 1B and C). The safe usage concentration was fixed at 15 µM in the following experiments. BBM also significantly inhibited the growth of H37Rv in THP-1-differentiated macrophages as determined by counting the CFUs at 48-hour post-infection (Fig. 1D). The experiment was repeated using mouse bone marrow–derived macrophages (BMDMs) to confirm these results, and similar findings were observed (Fig. 1E through G). Therefore, these data demonstrate that BBM enhances the bacteriostatic ability of macrophages against mycobacteria.

### Berbamine suppresses intracellular mycobacterial growth in macrophages by enhancing autophagy

**Fig 2:**
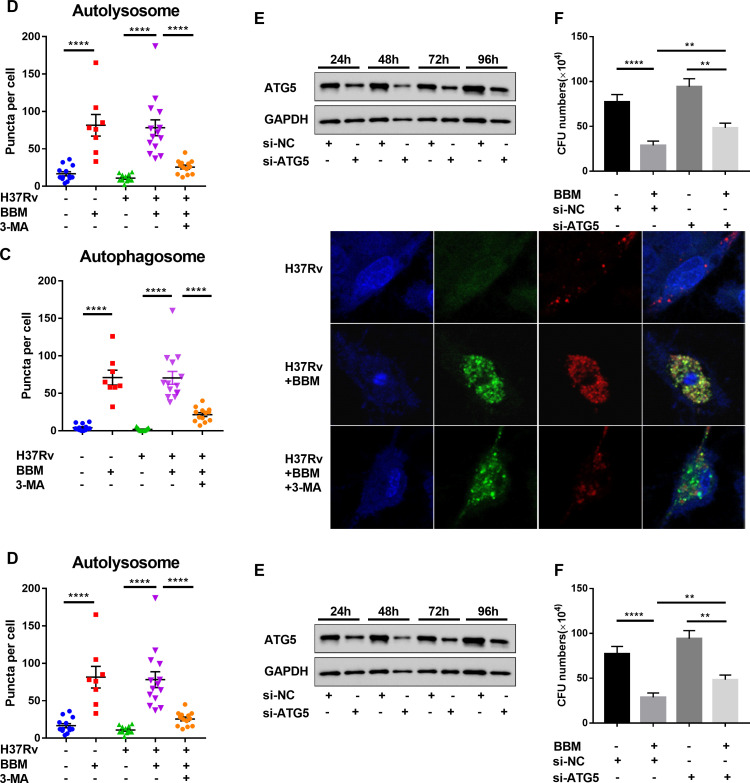
Berbamine treatment increased the expression level of LC3 and the formation of autophagosomes and autolysosomes in THP-1 cells. (**A**) PMA-differentiated THP-1 cells were infected with *M. tuberculosis* H37Rv (MOI 10:1) for 24 hours in the presence or absence of berbamine (15 µM) and 3-MA (5 mM). The LC3 protein level was analyzed by western blot. (**B**) mRFP-GFP-LC3 reporter THP-1 cells were differentiated by PMA and infected with *M. tuberculosis* H37Rv (MOI 10:1) with or without berbamine (15 µM) and 3-MA (5 mM) for 24 hours. Representative confocal microscopy images are shown (bars, 10 µm). The autophagosome puncta (yellow) per cell (**C**) and the autolysosome puncta (red) per cell (**D**) were calculated. (**E**) The knockdown efficiency of ATG5 siRNA at different time points after transfection was measured by western blot; scrambled siRNA was used as a negative control. (**F**) CFU counts from THP-1 cells in the presence or absence of berbamine (15 µM) and small interfering RNA of ATG5 (siATG5) after infection with *M. tuberculosis* H37Rv (MOI 10:1) for 48 hours. Data represent means ± SDs for three independent experiments. One-way ANOVA was performed in (C, D, and F). **P < 0.01; ****P < 0.0001.

**Fig 3:**
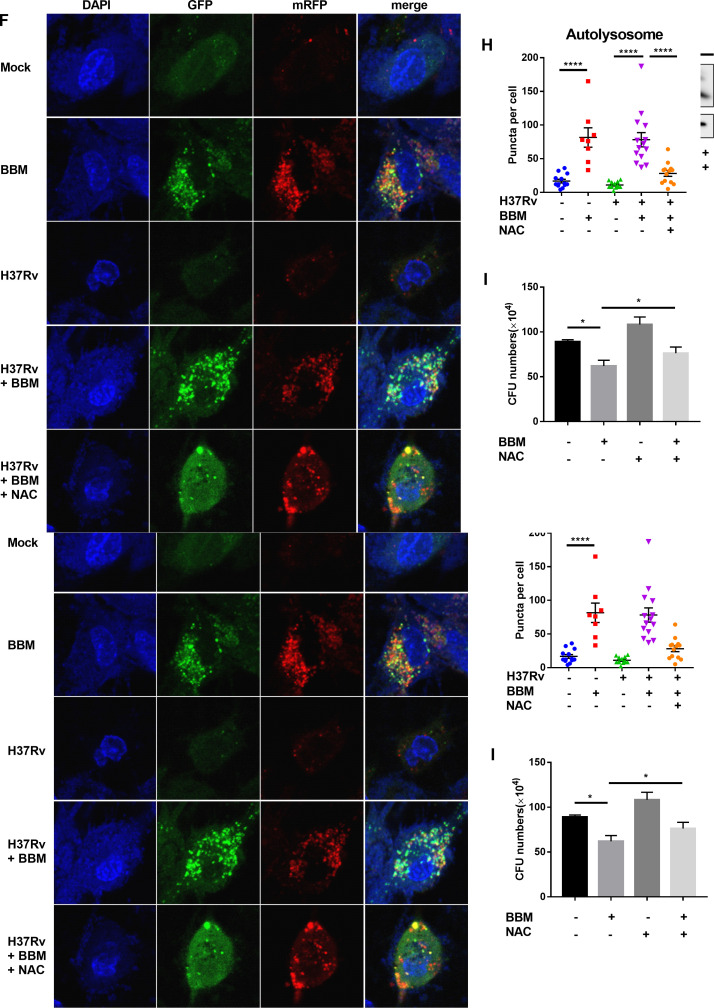
Berbamine-induced ROS further regulated autophagy in mycobacteria-infected THP-1 cells. PMA-differentiated THP-1 cells were infected with *M. tuberculosis* H37Rv (MOI 10:1) for 24 hours in the presence or absence of berbamine (15 µM), and cROS and mROS levels were detected using apocynin (10 µM) and MitoSOX (5 µM) probes. Representative flow cytometry images of cROS and mROS were captured (**A and C**). ROS’s mean fluorescence intensity (MFI) was analyzed by flow cytometry (**B and D**). Western blot of LC3 protein level from *M. tuberculosis* H37Rv-infected THP-1 cells with or without berbamine (15 µM) and NAC (10 mM) treatment (**E**). mRFP-GFP-LC3 reporter THP-1 cells were differentiated by PMA and infected with *M. tuberculosis* H37Rv (MOI 10:1) with or without berbamine (15 µM) and NAC (10 mM) for 24 hours. Representative confocal microscopy images are shown (bars, 10 µm) (**F**). The autophagosome puncta (yellow) per cell (**G**) and the autolysosome puncta (red) per cell (**H**) were calculated. (**I**) CFU counts from THP-1 cells in the presence or absence of berbamine (15 µM) and NAC (10 mM) after infection with *M. tuberculosis* H37Rv (MOI 10:1) for 48 hours. Data represent means ± SDs for three independent experiments. One-way ANOVA was performed in (**G–I**). *P < 0.05; ***P < 0.001; ****P < 0.0001.

To determine the mechanisms by which BBM inhibits intracellular survival of mycobacteria, BBM-treated infected cells were evaluated for autophagy, ROS production, and apoptosis induction. BBM treatment significantly increased autophagy (Fig. 2) and ROS production (Fig. 3) but had no impact on apoptosis in macrophages (Fig. S1). Autophagy is one of the most important mechanisms for macrophages to inhibit the growth of Mtb (6, 7). Therefore, the status of BBM on autophagy induction was investigated. BBM treatment significantly increased autophagic flux in macrophages (Fig. 2). LC3 is conventionally regarded as a marker of autophagy. A cytosolic form of LC3 (LC3-I) is conjugated to phosphatidylethanolamine to form LC3-phosphatidylethanolamine conjugate (LC3-II), which is recruited to autophagosomal membranes (8). In the western blot assay, BBM treatment markedly increased the conversion of LC3-I to LC3-II (Fig. 2A). To further explore the role of BBM in inducing autophagy, chloroquine (CQ), which inhibits autophagic flux by decreasing autophagosome–lysosome fusion (9), was used to block autophagy induced by BBM. The cumulative amount of LC3-II increased after using CQ in BBM-treated macrophages (Fig. S2), indicating that the increased accumulation of LC3-II promoted by BBM is not due to the inhibition of downstream autophagic flow. Another autophagy inhibitor, 3-methyladenine (3-MA), inhibits the activity of PI3-kinase, blocks the autophagosome’s formation and autophagic vacuoles (10), and was also used to block autophagy in the presence of BBM. 3-MA significantly inhibited BBM-induced autophagy as determined by immunoblotting of LC3-I to LC3-II conversion (Fig. 2A).

To further examine the status of BBM in autophagic flux, a THP-1 cell population stably expressing a tandem mRFP-GFP-LC3 fusion protein was established and used to visualize and distinguish autophagosome puncta (GFP^+^ mRFP^+^, yellow) and autolysosome puncta (GFP^−^ mRFP^+^, red). Under normal conditions, the mRFP-GFP-LC3 was present in the cytoplasm and showed a uniform distribution. When the cells underwent autophagy, the mRFP-GFP-LC3 fusion protein was translocated to the autophagosomal membrane, and the spots formed by the aggregation of red and green fluorescence were observed under a confocal microscope (11). Consistent with the above results, a significant increase of autophagosome and autolysosome-formed puncta was observed in BBM-treated macrophages, while 3-MA treatment reduced the formation of these BBM-induced LC3 puncta (Fig. 2B through D).

Next, to confirm the key function of autophagy in Mtb clearance with BBM treatment, ATG5, a crucial protein in autophagy initiation and processing (12), was knocked down by siRNA before BBM treatment. Transient transfection by siRNA was observed to sustain the effect of knockdown for 96 hours (Fig. 2E). Subsequently, a CFU assay to detect the survival of Mtb strain H37Rv in ATG5-knockdown THP-1 cells showed that ATG5 knockdown partially abolished BBM-mediated inhibition of intracellular growth of Mtb (Fig. 2F). The above results indicated the positive effect of BBM on autophagic flux for suppressing intracellular mycobacterial growth *in vitro*.

### Berbamine-induced autophagy is associated with ROS production

To determine the mechanisms by which BBM reduced intracellular survival of Mtb, the effect of BBM on the induction of intracellular ROS production during Mtb infection was monitored. ROS is a positive mechanism for killing intracellular Mtb by macrophages (13). The level of cROS was measured by the fluorescent probe apocynin, and mROS was measured by the red fluorescent dye MitoSOX. THP-1-differentiated macrophages were pretreated and maintained with 15 µM BBM for 24 hours, then were infected with H37Rv, and analyzed by flow cytometry at 24-hour post-infection, respectively. Both cROS and mROS production were significantly elevated in H37Rv-infected macrophages on treatment with BBM (Fig. 3A through D). Given that ROS could oxidize autophagy-associated proteins and facilitate the formation of autophagosomes (14), the effect of ROS on BBM-induced autophagy was tested. *N*-acetyl-L-cysteine (NAC), a powerful antioxidant, was added to the BBM-treated macrophages and abolished the induction of autophagy by BBM. Western blot showed that NAC treatment markedly inhibited the conversion of LC3-I to LC3-II (Fig. 3E). To confirm these results, the puncta of autophagosomes and autolysosomes were detected. Consistent with western blot, NAC treatment significantly inhibited the increased formation of autophagosome and autolysosome puncta by BBM (Fig. 3F through H). The CFU counts from lysed THP-1 cells infected with H37Rv were also increased by NAC treatment compared with the group treated by BBM alone. These findings suggested that BBM induced ROS production in Mtb-infected macrophages and the increased ROS production further promoted autophagy.

### Berbamine treatment increases intracellular Ca^2+^ concentration in mycobacteria-infected macrophages and initiates autophagy

**Fig 4:**
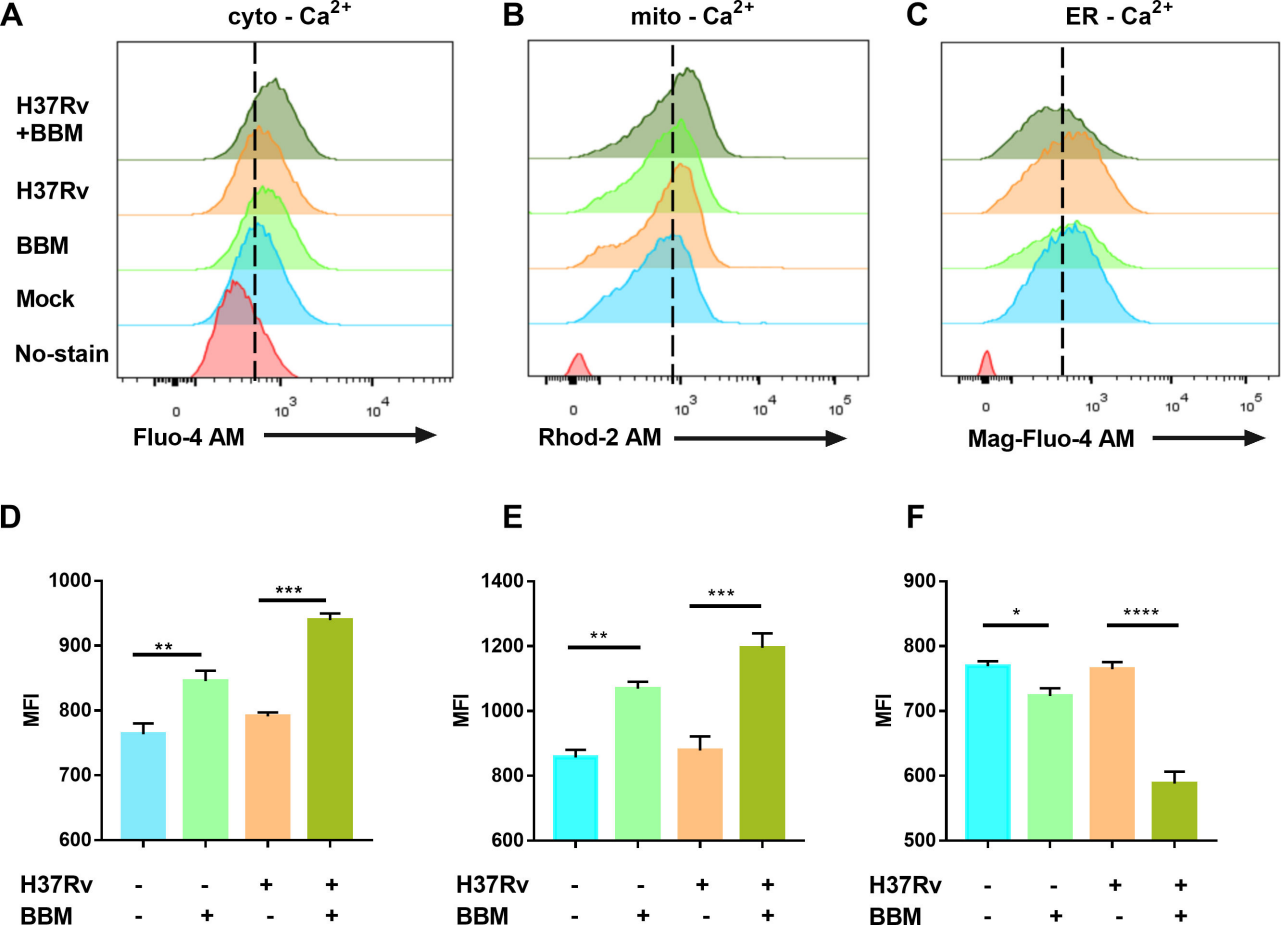
Berbamine treatment increased cytoplasmic Ca^2+^ (cyto-Ca^2+^) and mitochondrial Ca^2+^ (mito-Ca^2+^) concentrations. PMA-differentiated THP-1 cells were infected with *M. tuberculosis* H37Rv (MOI 10:1) for 24 hours in the presence or absence of berbamine (15 µM), and cyto-Ca^2+^, mito-Ca^2+^, and ER-Ca^2+^ levels were detected using Fluo-4 AM (5 µM), Rhod-2 AM (5 µM), and Mag-Fluo-4 AM (5 µM) probes, respectively. Representative flow cytometry images of cyto-Ca^2+^, mito-Ca^2+^, and ER-Ca^2+^ were captured (**A–C**). The mean fluorescence intensity (MFI) of Ca^2+^ was analyzed by flow cytometry (**D–F**). Data represent means ± SDs for three independent experiments. One-way ANOVA was performed in (**D–F**). ***P < 0.001; ****P < 0.0001.

Since Ca^2+^ signaling is essential for Mtb infections (15) and BBM is considered a calcium-channel blocker (16), the effect of BBM on intracellular Ca^2+^ concentration after Mtb infection was examined. Significant increases in cytoplasmic Ca^2+^ (cyto-Ca^2+^) (Fig. 4A and B) and mitochondrial Ca^2+^ (mito-Ca^2+^) (Fig. 4D and E) concentrations were detected in BBM-treated macrophages post-infection. Through searching literature, we found two other calcium-channel blockers, flunarizine and verapamil, which were also reported to upregulate macrophage calcium levels (17). We speculate that the mechanism of calcium-channel blockers differs between macrophages and excitable cells like heart and skeletal muscle cells. BBM was reported to inhibit the extracellular Ca^2+^ influx induced by KCl but did not affect intracellular resting Ca^2+^ concentration (18). Therefore, an experiment was conducted to explore whether the BBM-induced increase in Ca^2+^ concentration was related to extracellular Ca^2+^. The increase in Ca^2+^ concentration induced by BBM was not significantly changed by the presence or absence of extracellular Ca^2+^ (Fig. S3). Moreover, the Ca^2+^ concentration in the endoplasmic reticulum (ER) was determined to be decreased by BBM treatment. The increasing Ca^2+^ concentration in the cytoplasm and mitochondria by BBM treatment may be from the release of the ER Ca^2+^ pool; however, the detailed molecular mechanism of this process requires further exploration.

**Fig 5:**
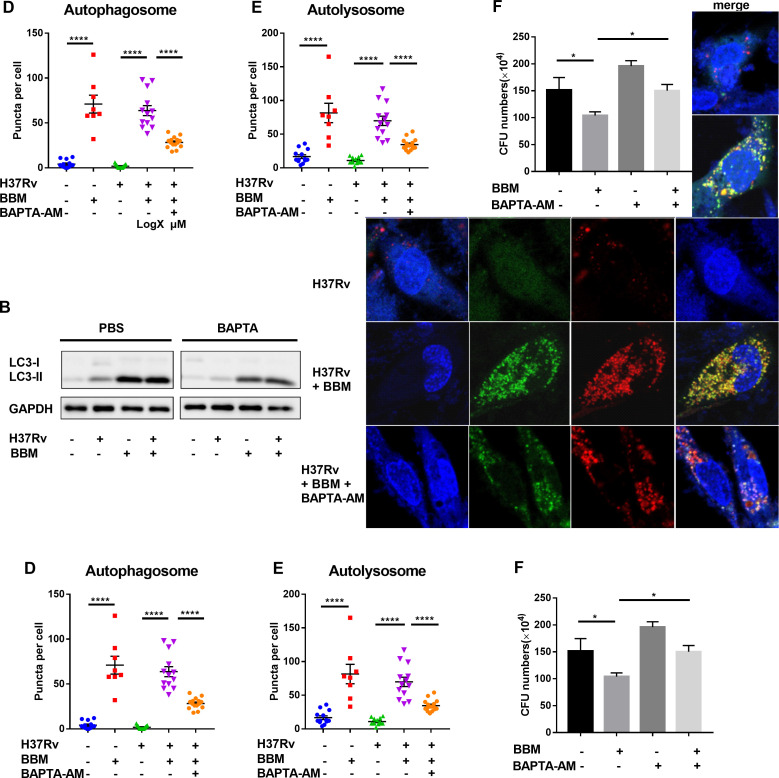
Ca^2+^-chelating agent BAPTA-AM inhibited the autophagy induced by berbamine. (**A**) Dose-dependent cytotoxicity and CC_50_ of BAPTA-AM in THP-1 cells. (**B**) Western blot of LC3 protein level from *M. tuberculosis* H37Rv-infected THP-1 macrophages with or without berbamine (15 µM) and BAPTA-AM (2 µM) treatment. (**C**) mRFP-GFP-LC3 reporter THP-1 cells were differentiated by PMA and infected with *M. tuberculosis* H37Rv (MOI 10:1) with or without berbamine (15 µM) and BAPTA-AM (2 µM) for 24 hours. Representative confocal microscopy images are shown (bars, 10 µm). The autophagosome puncta (yellow) per cell (**D**) and the autolysosome puncta (red) per cell (**E**) were calculated. (**F**) CFU counts from THP-1 cells in the presence or absence of berbamine (15 µM) and BAPTA-AM (2 µM) after infection with *M. tuberculosis* H37Rv (MOI 10:1) for 48 hours. Data represent means ± SDs for three independent experiments. One-way ANOVA was performed in (**D–F**). *P < 0.05; ****P < 0.0001.

Ca^2+^ is reported to be an activator of autophagy (19). Therefore, the potential association of BBM-induced Ca^2+^ with autophagy was explored. BAPTA-AM, a fast and potent intracellular Ca^2+^-chelating agent with a CC_50_ of 3.626 µM in THP-1 cells (Fig. 5A), was added to the BBM-treated macrophages. BAPTA-AM treatment markedly suppressed the conversion of LC3-I to LC3-II, as shown by western blot assay (Fig. 5B). Confocal microscopy observations of autophagosomes and autolysosomes and results of a CFU assay were consistent with the western blot result (Fig. 5C through F). Together, these data indicated that the BBM-induced increase in intracellular Ca^2+^ facilitated the development of autophagy.

### Berbamine-induced increase in intracellular Ca^2+^ is regulated by ROS

**Fig 6:**
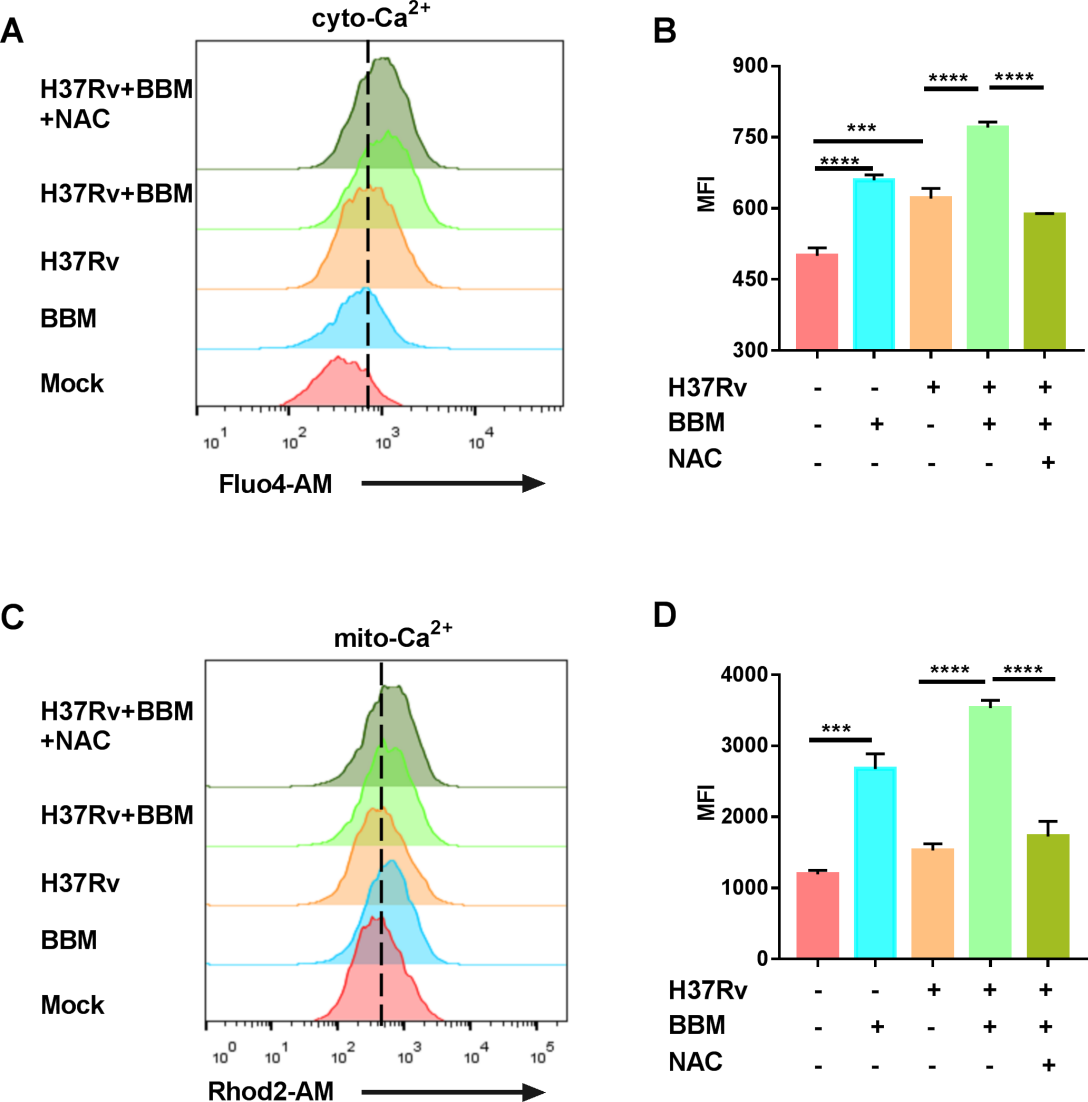
Inhibiting ROS could downregulate the Ca^2+^ concentration induced by berbamine. PMA-differentiated THP-1 cells were infected with *M. tuberculosis* H37Rv (MOI 10:1) for 24 hours in the presence or absence of berbamine (15 µM) and NAC (10 mM). Representative flow cytometry images of cyto-Ca^2+^ and Mito-Ca^2+^ were captured (**A and C**). The mean fluorescence intensity (MFI) of Ca^2+^ was analyzed by flow cytometry (**B and D**). Data represent means ± SDs for three independent experiments. One-way ANOVA was performed in (**B and D**). ***P < 0.001; ****P < 0.0001.

As BBM treatment was shown to increase intracellular ROS production and Ca^2+^ concentration, both of which promoted the processing of autophagy to control Mtb growth in macrophages, the association between ROS and Ca^2+^ concentration induced by BBM was further examined. It has been reported that ROS, directly and indirectly, affects Ca^2+^ transport in the plasma membrane, ER, and mitochondria (20). Thus, the determination of whether the ROS induced by BBM affected the Ca^2+^ concentration in Mtb-infected macrophages was conducted. NAC treatment alleviated the ability of BBM to induce increases in the concentration of cyto- and mito-Ca^2+^ (Fig. 6A through D). In addition, we also tested whether the ROS level was regulated by Ca^2+^ signaling. Surprisingly, BAPTA-AM treatment did not affect the intracellular ROS production induced by BBM (Fig. S4). These results implied that BBM-induced ROS production regulated the Ca^2+^ release in Mtb-infected macrophages.

### Berbamine treatment enhances the host defense against drug-resistant TB in macrophages

**Fig 7:**
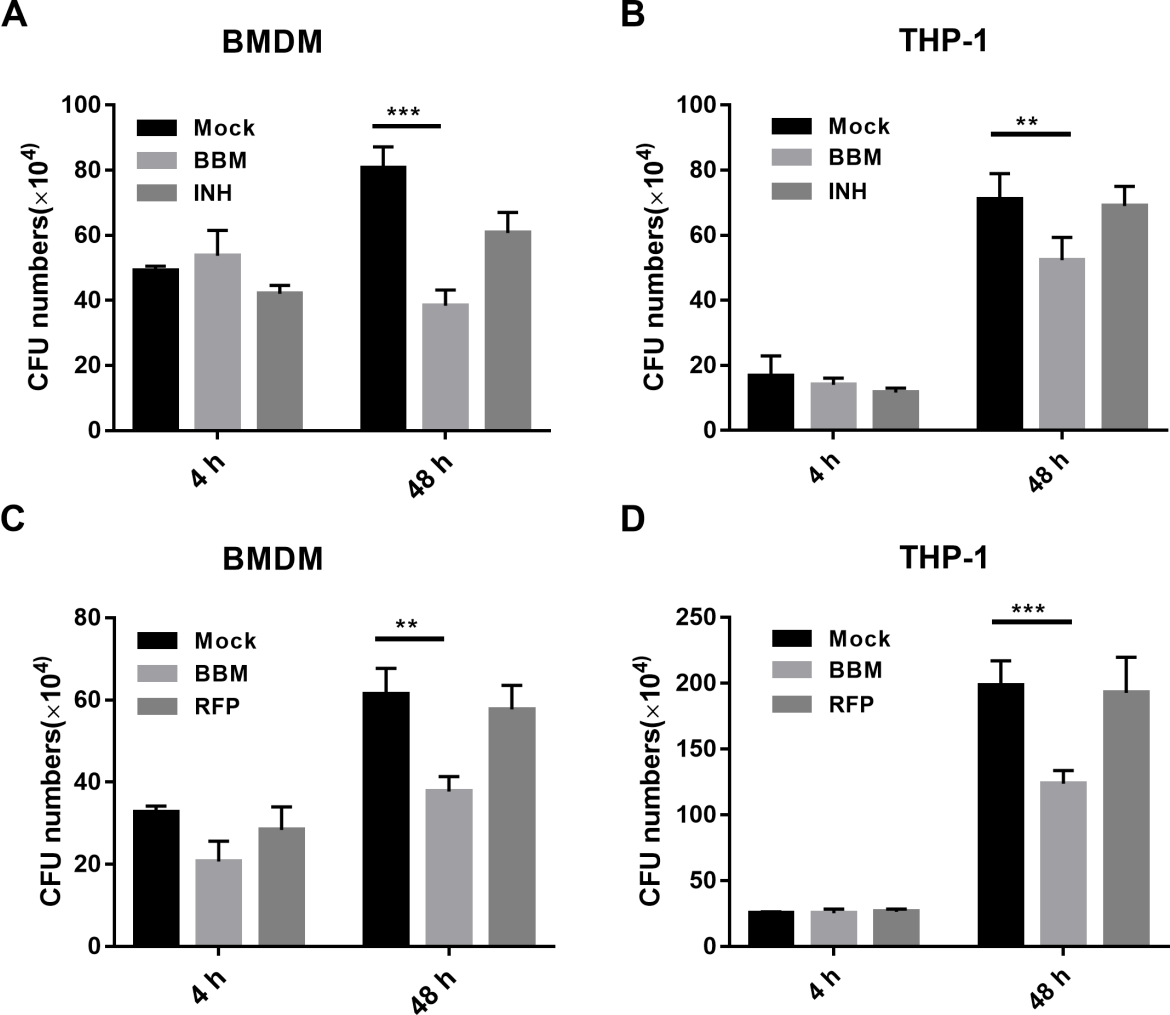
Berbamine suppressed drug-resistant tuberculosis growth within THP-1 cells and bone marrow–derived macrophages (BMDMs). PMA-differentiated THP-1 cells and BMDMs were pretreated with berbamine (15 µM) for 24 hours and then infected with INH-resistant tuberculosis (**A and B**) or RFP-resistant tuberculosis (**C and D**) for 48 hours before being lysed and plated on 7H10 plates. INH (0.1 µg/mL) and RFP (0.1 µg/mL) were added as control. The bactericidal activity in BMDMs (**A and C**) and THP-1 cells (**B and D**) was assessed by determining the CFU of the intracellular drug-resistant bacteria. Data represent means ± SDs for three independent experiments. Two-way ANOVA was performed in (**A and B**). **P < 0.01; ***P < 0.001.

Except for the standard virulence strain H37Rv, the effect of BBM on drug-resistant TB in macrophages was determined. As measured by CFU assay, BBM treatment inhibited drug-resistant Mtb in mouse BMDMs and THP-1 cells, whereas the frontline anti-TB drug isonicotinic acid hydrazide (INH, also known as isoniazid) (0.1 µg/mL) and rifampicin (RFP, 0.1 µg/mL) did not affect the growth of drug-resistant Mtb (Fig. 7A through D). These results indicated that BBM could enhance the clearance of drug-resistant Mtb and potentially be used in the therapy of MDR-TB and XDR-TB.

## DISCUSSION

New treatment strategies for TB are urgently needed due to the emergence of drug-resistant TB. Since the safety of FDA-approved drugs is recognized, repurposing such medicines for anti-TB therapy is a promising area of research in the fight against TB. In the current study, BBM, a natural compound from *B. amurensis* used in traditional Chinese medicine, significantly inhibits Mtb growth in human macrophages.

Autophagy is critical for maintaining intracellular homeostasis and defending against infectious pathogens, including Mtb (21). A previous study demonstrated that BBM protects the heart from ischemia/reperfusion (I/R) injury through the modulation of autophagy, characterized by an increased LC3-II level and GFP-LC3 puncta (22). BBM induced autophagy of human colon cancer cells, triggering autophagic vesicles’ development (23). BBM also improved the impaired autophagic flux in H_2_O_2_-induced senescent cells by activating AMP-activated protein kinase (AMPK) pathways (24), and AMPK activation negatively regulated the mechanistic target of rapamycin kinase (mTOR) pathway (25). BBM induced restoration of autophagic flux via the SIRT1/AMPK signaling axis in high-fat-diet rats (26). Consistent with this research, BBM treatment in the current study significantly increased the autophagy flux in Mtb-infected macrophages, as characterized by increased conversion of LC3-I to LC3-II. BBM was further shown to enhance the formation of autophagosome and autolysosome puncta, indicating that BBM promoted the elimination of intracellular Mtb via autophagosome synthesis and lysosome degradation.

ROS and reactive nitrogen species are generated immediately in macrophages after the recognition of invading bacteria (13). It is well established that berberine modulates the generation of ROS in various disorders, including diabetes, cancers, and various inflammatory conditions (27). BBM increased the intracellular ROS level by downregulating antioxidative genes such as *Nrf2*, *HO-1*, *SOD2*, and *GPX-1* to suppress the progression of bladder cancer (28), while a novel synthetic BBM derivative, BBMD3, increased the production of ROS in osteosarcoma cells (29). In the present study, ROS production was also increased by BBM treatment in Mtb-infected macrophages. Because the generation of ROS promotes the recruitment of LC3 to the phagosome, facilitating phagosome–lysosome fusion (30), the relationship between ROS and autophagy in BBM-treated macrophages was subsequently explored. The ROS scavenger NAC could block the increase in autophagy flux and the formation of autophagosome and autolysosome puncta in Mtb-infected macrophages, leading to the conclusion that BBM induced ROS production and further initiated autophagy to suppress intracellular Mtb growth.

BBM is considered to be a calcium-channel blocker, and calcium is known to play an important role in TB pathogenesis. However, in the current study, BBM induced intracellular calcium accumulation in macrophages. Two other calcium-channel blockers, flunarizine and verapamil, were also reported to upregulate macrophage calcium levels (17). We speculate that the mechanism of calcium-channel blockers differs between macrophages and excitable cells like heart and skeletal muscle cells. Next, since elevated cytosolic calcium concentrations also promote the autophagic process (31), the impact of the BBM-induced increase in calcium concentration on autophagy was evaluated. The increase in calcium concentration by BBM treatment facilitated the development of autophagy in Mtb-infected macrophages, while the calcium-chelating agent BAPTA-AM suppressed the upregulation of autophagy by BBM.

Furthermore, ROS are reported to affect ER calcium homeostasis via inositol 1,4,5-trisphosphate receptor (32) and ryanodine receptor channels (33). In the current study, the calcium concentration induced by BBM in Mtb-infected macrophages could be inhibited by the antioxidant NAC, indicating that the BBM-induced increase in intracellular calcium is regulated by ROS. However, the exact signal pathway from ROS to calcium and autophagy during BBM treatment requires further exploration.

A vital role of HDT is against drug-resistant Mtb, consequently, the effect of BBM on drug-resistant Mtb was also evaluated. BBM effectively inhibited the growth of INH-resistant and RFP-resistant Mtb in macrophages. BBM is an over-the-counter drug considered safe and effective for the general public without a prescription (34). Findings from the current study indicate that BBM may have the potential as a therapeutic against drug-resistant Mtb.

In summary, BBM was shown to suppress the growth of intracellular mycobacteria potently, and this effect was mediated by BBM enhancing the calcium concentration, ROS, and autophagy in infected macrophages. These findings suggest that BBM may be considered an HDT candidate for TB treatment, and further *in vivo* investigations are warranted.

## SUPPLEMENTAL MATERIAL

Figure S1 mbio.00272-23-s0001.tif Berbamine did not affect the apoptosis of mycobacterium-infected macrophages.

Figure S2 mbio.00272-23-s0002.tif Chloroquine blocked the autophagic flux induced by berbamine treatment.

Figure S3 mbio.00272-23-s0003.tif Berbamine increased cytoplasmic Ca^2+^ (cyto-Ca^2+^) and mitochondrial Ca^2+^ (mito-Ca^2+^) concentrations without affecting by extracellular Ca^2+^.

Figure S4 mbio.00272-23-s0004.tif Ca^2+^-chelating agent BAPTA-AM did not affect the ROS level induced by berbamine.

Supplemental figure legends mbio.00272-23-s0005.docx Legends to Fig. S1 to S4.

ASM does not own the copyrights to Supplemental Material that may be linked to, or accessed through, an article. The authors have granted ASM a non-exclusive, world-wide license to publish the Supplemental Material files. Please contact the corresponding author directly for reuse.
