# Supplementary figures and images for: Berbamine promotes macrophage autophagy to clear Mycobacterium tuberculosis by regulating the ROS/Ca2+ axis
Source: mBio. 2023 Jun 29;14(4):e00272-23. doi: 10.1128/mbio.00272-23 (PMC10470588; doi:10.1128/mbio.00272-23)

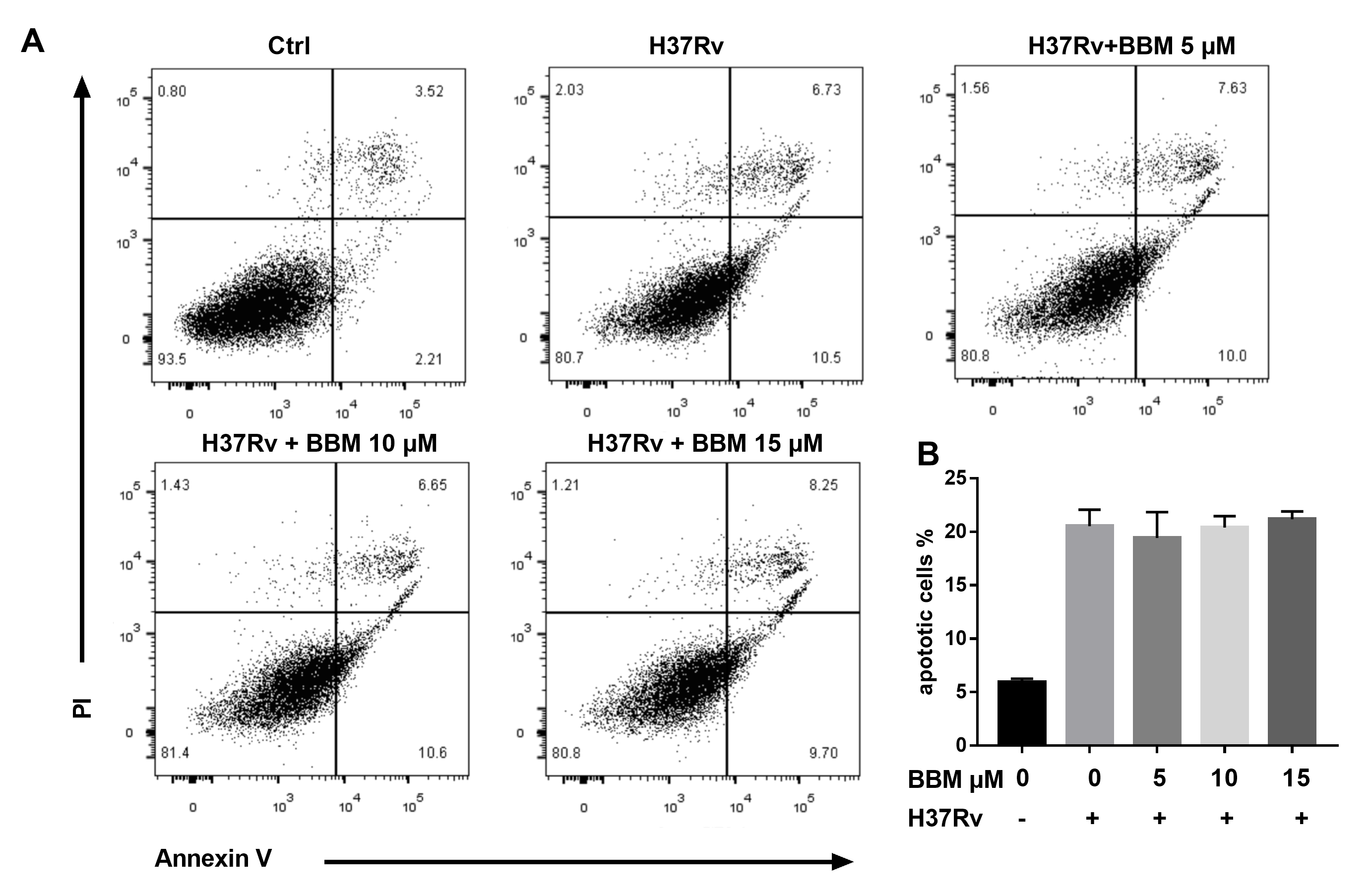

Supplement: Figure S1 — Berbamine did not affect the apoptosis of mycobacterium-infected macrophages. [file mbio.00272-23-s0001.tif]

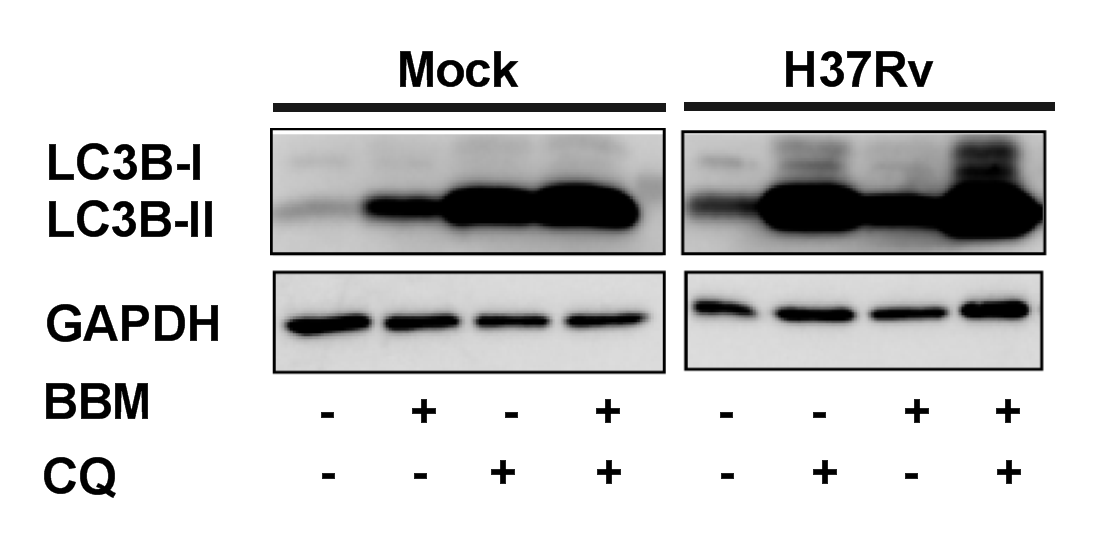

Supplement: Figure S2 — Chloroquine blocked the autophagic flux induced by berbamine treatment. [file mbio.00272-23-s0002.tif]

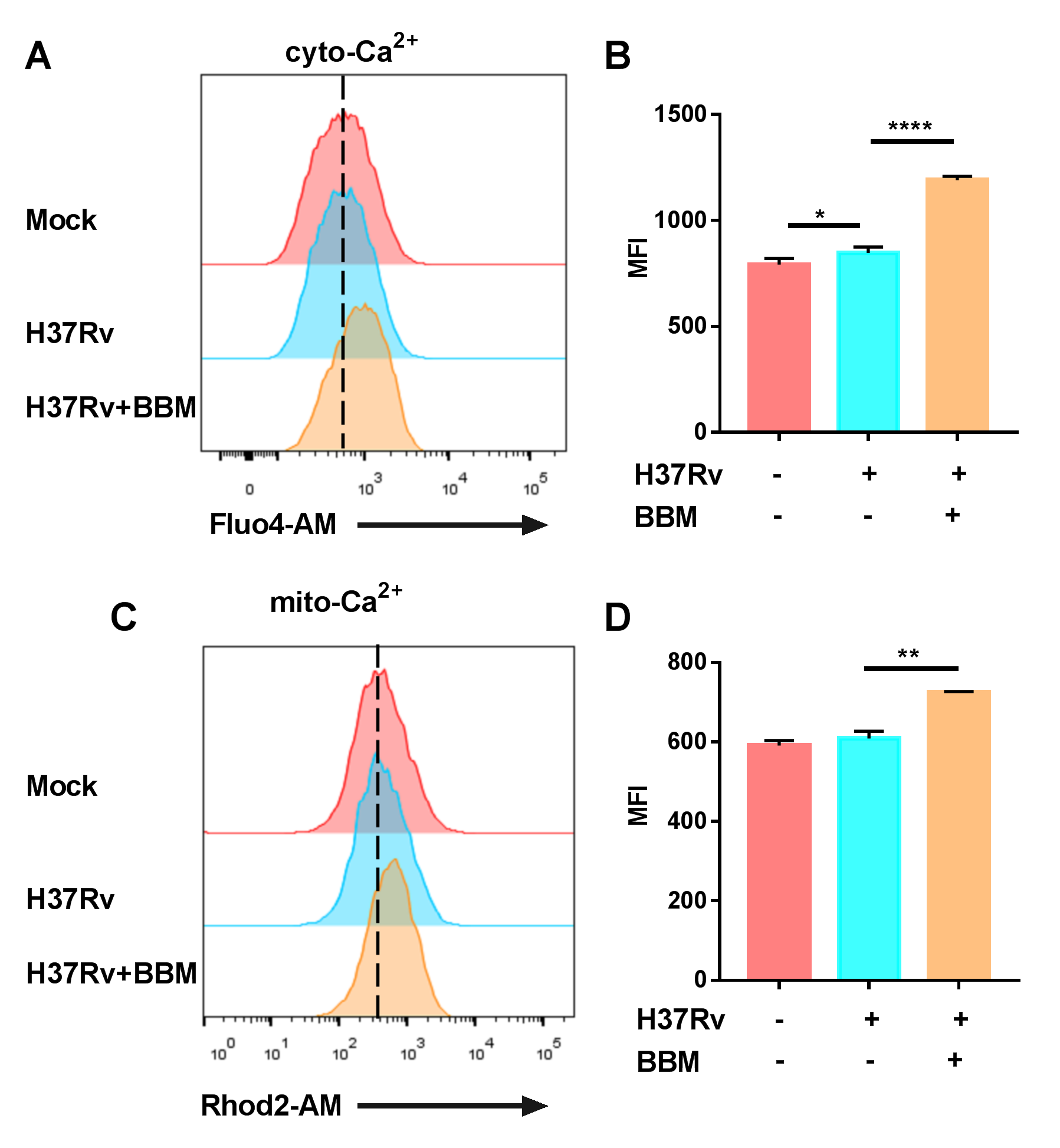

Supplement: Figure S3 — Berbamine increased cytoplasmic Ca2+ (cyto-Ca2+) and mitochondrial Ca2+ (mito-Ca2+) concentrations without affecting by extracellular Ca2+. [file mbio.00272-23-s0003.tif]

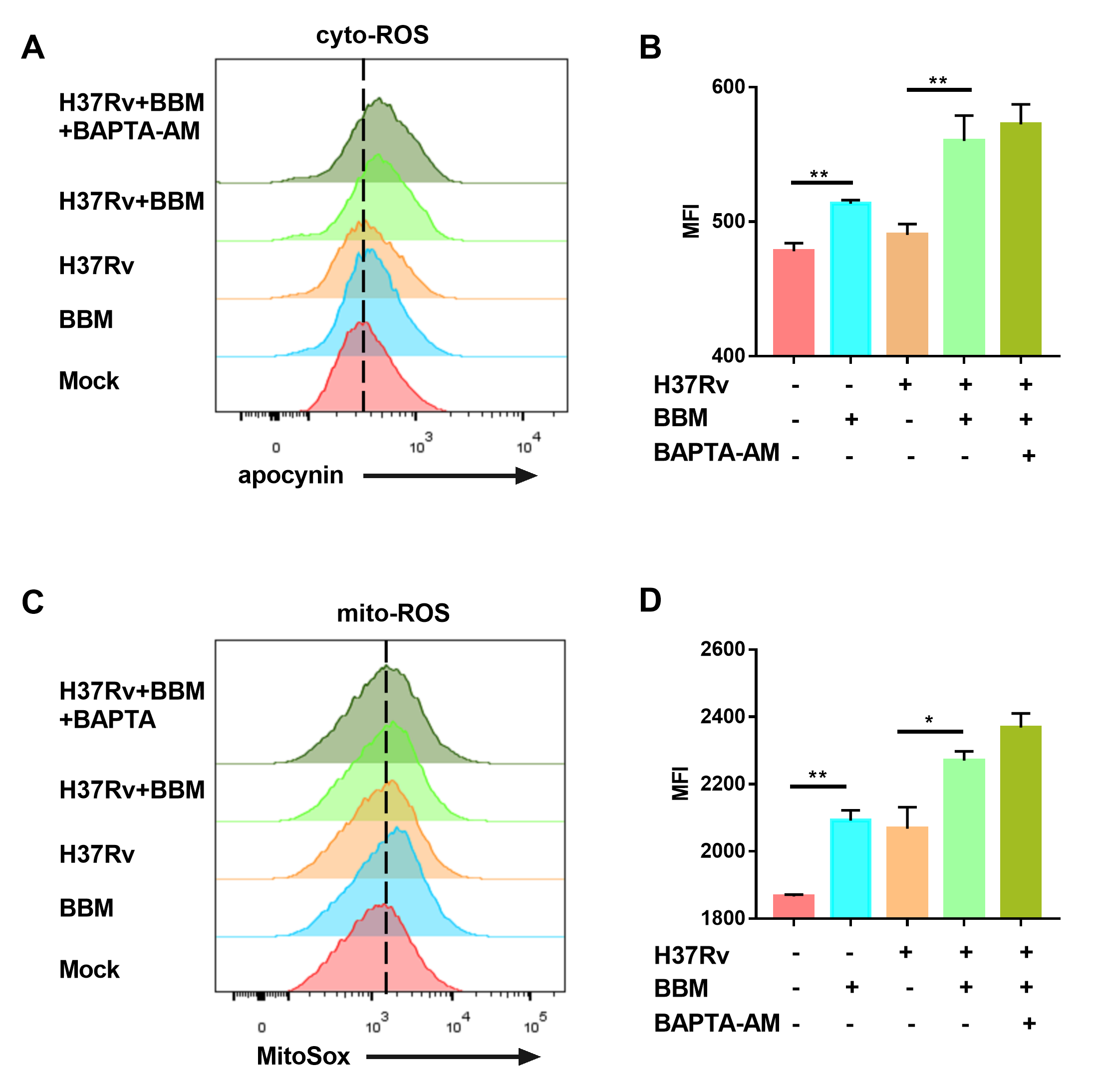

Supplement: Figure S4 — Ca2+-chelating agent BAPTA-AM did not affect the ROS level induced by berbamine. [file mbio.00272-23-s0004.tif]
