# Supplementary material for: Berbamine promotes macrophage autophagy to clear Mycobacterium tuberculosis by regulating the ROS/Ca2+ axis
Source: mBio. 2023 Jun 29;14(4):e00272-23. doi: 10.1128/mbio.00272-23 (PMC10470588; doi:10.1128/mbio.00272-23)
Supplement: Supplemental figure legends — Legends to Fig. S1 to S4. [file mbio.00272-23-s0005.docx]

**Supplementary Figure 1. Berbamine did not affect the apoptosis of mycobacteria-infected macrophages.** PMA-differentiated THP-1 cells were infected with *M. tuberculosis* H37Rv for 24 h, and apoptosis was detected by Annexin V and propidium iodide (PI) staining. **(A)** Representative flow cytometry images of apoptotic cells. **(B)** Percentage of apoptotic cells in the presence of the indicated dose of berbamine; the percentages of apoptotic cells were calculated by Flow Jo software. Data represent three independent experiments with similar results and are presented as means±SD. One-way ANOVA was performed in **(B)**.

**Supplementary Figure 2. Chloroquine blocked the autophagic flux induced by berbamine treatment.** PMA-differentiated THP-1 cells were infected with *M. tuberculosis* H37Rv (MOI 10:1) for 24 h in the presence or absence of berbamine (15 μM) and chloroquine (CQ) (50 μM). The LC3 protein level was analyzed by Western blot. Representative data from two independent experiments with similar results are shown.

**Supplementary Figure 3. Berbamine increased cytoplasmic Ca^2+^ (cyto-Ca^2+^) and mitochondrial Ca^2+^ (mito-Ca^2+^) concentrations without affecting** **by** **extracellular Ca^2+^.** Berbamine-treated THP-1 cells were cultured in a Ca^2+^-free medium, and cyto-Ca^2+^ and mito-Ca^2+^ levels were detected 24 h after infection of the cells with *M. tuberculosis* H37Rv. Representative flow cytometry images of cyto-Ca^2+^ and mito-Ca^2+^ were captured **(A, C).** The mean fluorescence intensity (MFI) of Ca^2+^ was analyzed by flow cytometry **(B, D)**. Data represent means±SD for three independent experiments. One-way ANOVA was performed in **(B, D)**. ** *P* < 0.01; **** *P* < 0.0001.

**Supplementary Figure 4.**  **Ca^2+^-chelating agent BAPTA-AM did not affect the ROS level induced by berbamine.** PMA-differentiated THP-1 cells were infected with *M. tuberculosis* H37Rv (MOI 10:1) for 24 h in the presence or absence of berbamine (15 μM) and BAPTA-AM (2 μM). Representative flow cytometry images of cyto-ROS and Mito-ROS were captured **(A, C).** The mean fluorescence intensity (MFI) of ROSs was analyzed by flow cytometry **(B, D)**. Data represent means±SD for three independent experiments. One-way ANOVA was performed in **(B, D)**. * *P* < 0.05; ** *P* < 0.01.
